# Ultrasonographic evaluation of the effects of the administration of neostigmine and metoclopramide on duodenal, cecal, and colonic contractility in Arabian horses: A comparative study

**DOI:** 10.14202/vetworld.2020.2447-2451

**Published:** 2020-11-16

**Authors:** Noha Abdallah Beder, Ahmed Atef Mourad, Mahmoud Allam Aly

**Affiliations:** 1 Department of Animal Medicine, Faculty of Veterinary Medicine, Damanhour University, Egypt; 2 Department of Surgery, Anesthesiology and Radiology, Faculty of Veterinary Medicine, University of Sadat City, Egypt; 3 Department of Animal Medicine and Infectious Diseases, Faculty of Veterinary Medicine, University of Sadat City, Egypt

**Keywords:** Arabian horse, intestinal contractility, metoclopramide, neostigmine, ultrasonography

## Abstract

**Background and Aim:**

As means for enhancing gastrointestinal (GI) motility, prokinetics have previously been evaluated in different breeds of horses with varying success. No previous studies were conducted to evaluate the effect of prokinetics on the intestinal tract of Arabian horses breed. Using B-mode ultrasound in the quantification of intestinal contractility, this study aimed to compare the impact of neostigmine and metoclopramide on the intestinal motility of healthy Arabian horses.

**Materials and Methods:**

Twenty-one clinically healthy Arabian horses were equally distributed into three groups. The control group was administered with 5 mL normal saline intramuscularly (IM). The second group was administered with neostigmine (0.044 mg/kg body weight [BW], IM), and the third group was administered with metoclopramide (0.25 mg/kg BW, IM). Duodenal, cecal, and colonic contractions were counted through ultrasonography for 3 min (pre-administration and 15, 30, 60, 120, and 180 min post-administration).

**Results:**

In the neostigmine group, a significant (p<0.05) increase in duodenal, cecal, and colonic contractions was observed 15 min post-administration compared with that in the control group (15±1.0, 11.33±1.53, and 12.33±2.31 vs. 11.0±2.0, 6.33±0.58, and 5.33±0.58 contractions per 3 min, respectively), continuing to 60 min post-administration for the duodenum and cecum and 120 min for colon and then returning to the normal; however, the metoclopramide group showed a significant (p<0.05) increase only in cecal and colonic contractions 60 min post-administration compared with the control group (11.0±1.0 and 12.33±0.58 vs. 6±1.0 and 5.67±0.58 contractions per 3 min, respectively), continuing until the end of the experiment. Excessive sweating, excitation, and straining were recorded following the administration of neostigmine, whereas no side effects were observed in the metoclopramide group.

**Conclusion:**

Neostigmine improves duodenal, cecal, and colonic contractions in healthy adult Arabian horses, whereas metoclopramide only improves cecal and colonic contractions. Metoclopramide appears to be safer and longer acting than neostigmine in the Arabian horse breed. Ultrasonography is a valuable noninvasive tool for the quantification of intestinal contractility. Future studies should consider the use of various dosages of metoclopramide and administration routes and investigate its impact on horses with GI transit disorders and inclusion in colic post-operative care.

## Introduction

Abnormalities of gastrointestinal (GI) motility are often a challenge in horses [1]. Duodenitis, peritonitis, electrolyte imbalances, endotoxemia, anesthesia, bowel ischemia, and intestinal distension are characterized by the loss of intestinal motility [2,3]. In addition, both post-operative reflux and post-operative ileus (POI) are life-threatening disorders of impaired intestinal motility that may occur in horses after colic surgery [4,5]. Prokinetics are effective agents in the treatment of diseases with diminished bowel motility and POI cases [6]. These agents enhance GI motility by increasing the frequency of intestinal contractions or making them stronger [7]. A variety of prokinetic agents has been used in horses with varying success. Neostigmine prolongs the activity of acetylcholine by delaying its breakdown at the synaptic junction [8]. Its prokinetic effect was previously studied in healthy ponies using scintigraphy [3] and *in vitro* testing of the contractility of intestinal smooth muscle strips of adult horses [9]. It is the most commonly selected prokinetic for the treatment of large colon impaction [10]. Conversely, metoclopramide has direct and indirect stimulating effects on cholinergic receptors, leading to increased acetylcholine release in the nerve endings of the GI tract and promotion of GI motility, as well as central and peripheral anti-dopaminergic effects [1,6]. Its prokinetic effect was previously evaluated in healthy adult thoroughbred horses using electrointestinography [6] and was also evaluated in a study of ponies with POI evaluating electromechanical activity [8]. Although it was successful in countering experimentally induced colic and POI (both naturally occurring and experimentally induced [11]), its efficacy in horses requires further investigation [1].

Although there are several methods to evaluate equine GI transit [12], abdominal ultrasound is the most practical tool in clinical situations [5,13-15]. It is a relatively easy and non-invasive tool for evaluating anatomical location, wall thickness, motility, and intestinal contents [16]. The equine intestinal motor activity has previously been evaluated using several techniques, including molecular biology, immunohistochemistry, tissue culture [12], and electrointestinography [6]; meanwhile, limited attempts have been made to quantify intestinal activity in horses using B-mode ultrasound [17]. Laus *et al*. [1] ultrasonographically evaluated intestinal hypomotility in Standardbred horses with a primary equine squamous gastric disease, whereas Lawson *et al*. [5] evaluated only equine duodenal motility after colic surgery.

To the best of our knowledge, no previous studies that evaluated the effect of prokinetics on the intestinal activity of Arabian horses were conducted. Hence, this study aimed to compare the impact of neostigmine and metoclopramide on the intestinal tract of healthy Arabian horses using ultrasound to quantify intestinal motor activity and monitor any adverse reactions.

## Materials and Methods

### Ethical approval

This study was approved by the Institutional Animal Ethics Committee, Animal Reproduction Research Institute, Egypt.

### Horses and management

Twenty-one adult, Egyptian Arabian horses (12 non-pregnant mares and nine stallions) in the period from March 2018 to June 2019 were used in this study. They were deemed clinically healthy without any GI tract disorders following physical, hematological, and biochemical examinations at the hospital of Animal Reproduction Research Institute, Egypt. They were aged 7-15 years old and weighed 300-450 kg. They were kept in tie stalls with daily turn-out in a paddock and were fed daily a diet of free choice grass hay and 1 kg concentrates with unlimited access to water.

### Study design

Twenty-one horses were distributed randomly into three groups (seven horses per group). The control group was administered with 5 mL of normal saline intramuscularly (IM). The neostigmine-treated group was administered with Neostigmine methyl sulfate^®^ 0.5 mg/mL (Amriya Pharm. Ind., Egypt), IM, at a dose rate of 0.044 mg/kg body weight (BW). The metoclopramide-treated group was administered with Primperan^®^ 10 mg/2 mL ampule (Sanofi-Aventis, Egypt), IM, at a dose rate of 0.25 mg/kg BW. Drug effect was evaluated in four trials with intervals of 1 week. One hour after feeding, the motility of the descending duodenum, cecum, and left ventral colon was evaluated through ultrasonography. The examination of behavioral patterns, the heart, and respiratory rates after drug administration was conducted during ultrasonography.

### Ultrasonographic examination

Trans-abdominal B mode ultrasonography was conducted using an ultrasound machine (Exagyne ECM Company, France) with a 3.5 MHz microconvex probe. The hair was removed, and the skin was cleaned with alcohol. Next, ultrasonic coupling gel was applied to achieve high-quality imaging. The positions of the descending duodenum, cecal body, and colon in each horse were identified by anatomic location and ultrasonographic appearance [1].

**Figure-1:**
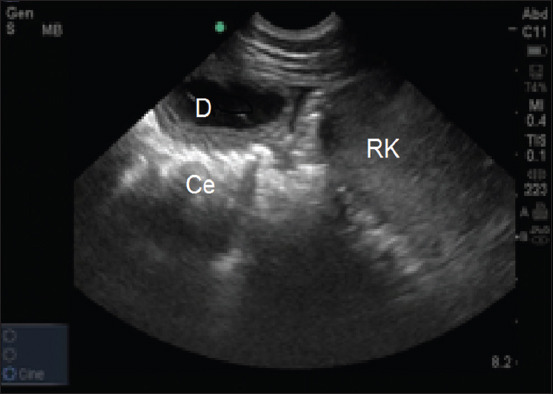
Sonogram of the duodenum (D) at the right 16^th^ ICS in an Arabian horse. Ce=Cecum with distal shadowing. RK=Right kidney. Dorsal is to the right of the image.

**Figure-2:**
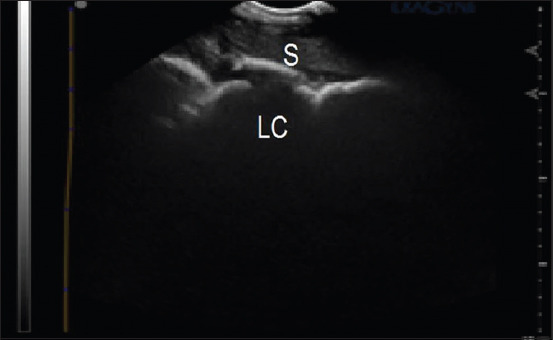
Sonogram of the left ventral colon with its sacculations (LC) in an Arabian horse. S=Spleen.

The duodenum was imaged ventral to the right kidney in the 15^th^ and 16^th^ intercostal spaces, along the line joining the olecranon and the tuber coxae. It appeared tubular, changing to circular when imaged in its short axis, with a hypoechoic-to-echogenic wall (Figure-1). The cecal body was scanned in the upper part of the right paralumbar region, whereas the left ventral colon was located ventromedial to the spleen; both appeared as mobile hypoechoic lines with an underlying hyperechoic gas echo. Their echoes were characterized by a large semi-curved, sacculated appearance (Figure-2).

The motility of the duodenum, cecum, and left ventral colon was evaluated, and the counting of contractions using ultrasonography was conducted for 3 min before treatment and at 15, 30, 60, 120, and 180 min post-administration for each anatomical location as previously described [17,18]. Duodenal motility was evaluated in the cross-sectional view by counting the number of circular contractions. Cecal contractions were assessed based on the moving of the cecal wall away from the transducer. Regarding the colon, changes in sacculations were evaluated.

### Statistical analysis

Data analyses were conducted using the statistical software program SPSS for Windows version 25 (SPSS Inc., Chicago, USA). Data were tested for normal distribution using the Shapiro–Wilk test. The data were normally distributed; therefore, the mean and standard deviation (SD) were calculated for each evaluated treatment at each time point. One-way analysis of variance with Tukey’s *post hoc* multiple comparison tests was used to determine statistically significant differences between the control group and the groups treated with drugs. All data were expressed as mean±SD. p≤0.05 was considered statistically significant.

## Results

Clinical examination of the horses showed normal findings. The mean body temperature was 37.5°C±0.11°C, and the respiratory and pulse rates were 12.9±0.69 breaths/min and 32±1.11 beats/min, respectively. Conjunctival mucous membranes, intestinal sounds, and rectal findings were all normal. The fecal examination for parasite eggs, sand, and blood was negative. Packed cell volume, total erythrocyte count, and hemoglobin were 39.2%±2.23%, 6.33±0.45 million/mm^3^, and 14.6±0.42 g/dL, respectively. The results of liver and renal function tests were within the normal range (aspartate aminotransferase, 116.0±7.23 U/L; gamma-glutamyl transferase, 16.10±2.10 U/L; blood urea nitrogen, 21.00±1.54 mg/dL; and creatinine, 1.18±0.06 mg/dL).

Tables-1-3 summarize the results of the contraction measurements for the duodenum, cecum, and colon, respectively. Regarding the duodenum, the neostigmine group significantly increased its contraction (p<0.05) 15 min on administration compared with those of the metoclopramide and control groups (15±1.0 vs. 11.67±1.53 and 11.0±2.0 contractions per 3 min, respectively). This increase in duodenal contraction persisted for 60 min post-administration and then decreased 120 min post-administration and for the following period. Meanwhile, the metoclopramide group did not show any significant change in duodenal contraction compared with the control group over the entire study period.

**Table-1:** Effect of neostigmine and metoclopramide on duodenal contractions.

Time/min	Neostigmine group	Metoclopramide group	Control group
0	12.33±0.58^a^	12.33±0.58^a^	11.67±1.53^a^
15	15.0±1.0^a^	11.67±1.53^b^	11.0±2.0^b^
30	19.33±1.15^a^	12±2.65^b^	11.7±0.6^b^
60	18.33±0.58^a^	10.67±1.53^c^	12.0±0.00^bc^
120	12.0±2.65^a^	10.90±1.0^a^	12.0±1.0^a^
180	11.0±1.0^a^	10.67±0.588^a^	11.7±0.6^a^

Values are means±SD; means with different superscripts in the same raw are significantly different (p<0.05)

**Table-2:** Effect of neostigmine and metoclopramide on cecal contractions.

Time/min	Neostigmine group	Metoclopramide group	Control group
0	6.67±0.58^a^	7.0±1.0^a^	7.0±1.0^a^
15	11.33±1.53^a^	7.0±1.0^bc^	6.33±0.58^c^
30	13.67±2.1^a^	7.0±1.0^bc^	6.67±0.58^c^
60	12.33±1.53^a^	11.0±1.0^ab^	6.00±1.00^c^
120	8.33±2.1^b^	14.67±0.58^a^	6.67±1.53^bc^
180	7.67±0.58^b^	14.0±1.0^a^	6.33±1.53^bc^

Values are means±SD; means with different superscripts in the same raw are significantly different (p<0.05)

**Table-3:** Effect of neostigmine and metoclopramide on colonic contractions.

Time/min	Neostigmine group	Metoclopramide group	Control group
0	5.33±0.58^a^	5.67±0.58^a^	4.67±0.58^a^
15	12.33±2.31^a^	7.33±1.53^bc^	5.33±0.58^c^
30	15.0±1.0^a^	8.67±3.1^b^	5.67±0.58^bc^
60	13.67±1.53^a^	12.33±0.58^ab^	5.67±0.58^c^
120	8.67±1.53^b^	11.67±0.58^a^	5.0±1.0^c^
180	7.0±1.0^ab^	8.0±1.0^a^	5.67±0.58^b^

Values are means±SD; means with different superscripts in the same raw are significantly different (p<0.05).

Similar to the duodenum, cecal contraction significantly increased (p<0.05) 15 min post-administration in the neostigmine group compared with those in the metoclopramide and control groups (11.33±1.53 vs. 7.0±1.0 and 6.33±0.58 contractions per 3 min, respectively). Furthermore, this increase persisted at 60 min post-administration and then decreased later. Cecal contraction significantly increased (p<0.05) in the metoclopramide group at 60 min post-administration compared with that in the control group (11.0±1.0 vs. 6.00±1.0 contractions per 3 min); however, this increase continued until the end of the experiment.

Colon contraction was higher (p<0.05) in the neostigmine group than in the metoclopramide and control groups at 15 min (12.33±2.31 vs. 7.33±1.53 and 5.33±0.58 contractions per 3 min, respectively). This significant change continued until 120 min post-administration relative to the control group. Meanwhile, colon contractions increased significantly (p<0.05) in the metoclopramide group compared with those in the control group at 60 min on administration (12.33±0.58 vs. 5.67±0.58 contractions per 3 min), continuing until the end of the experiment. Besides the prokinetic effect, the animals in the neostigmine group exhibited symptoms that suggest the development of abdominal pain, sweating, restlessness, excitement, and straining 15 min after injection; in the metoclopramide group, no adverse reactions were observed during the experiment.

## Discussion

Despite the range of prokinetic drugs available to equine practitioners, there is still controversy regarding the most suitable one for each individual clinical case [19]. In this study, neostigmine administration was associated with a rapid and significant increase in duodenal, cecal, and colonic contractility 15 min post-administration followed by a decline at 120 min. Wong *et al*. [19] stated that neostigmine increased the myoelectric activity of the ileum, cecum, and colon as well as stimulated the motility of pelvic flexure and decreased the cecal emptying time in clinically normal ponies. Nieto *et al*. [9] also observed a dose-dependent increase in the contractile amplitude of jejunum and pelvic flexure muscle strips of adult horses. However, Delesalle *et al*. [11] stated that neostigmine inhibited the motility of the proximal part of the GI tract. The rapid effect of neostigmine could be due to the activation of muscarinic receptors that are concentrated along the GI tract. The shorter effect observed for neostigmine than for metoclopramide could be due to muscle fatigue following severe contraction associated with the cholinergic side effects of neostigmine. Signs of abdominal discomfort were observed as adverse reactions shortly after the injection of neostigmine similar to those previously reported by Delesalle *et al*. [11] in horses and Wong *et al*. [19] in clinically normal ponies.

Metoclopramide is one of the benzamide prokinetic drugs acting as a 5-hydroxytryptamine 4 (5-HT_4_) receptor agonist [20-22] and a 5-HT_3_ and dopamine (D_2_) receptor antagonist [6]. The distribution of 5-HT_4_ receptors in the equine gut and the efficacy of different selective 5-HT_4_ agonists have previously been investigated. In *in vitro* studies, the 5-HT_4_ receptor agonist tegaserod increased the contractility of isolated smooth muscle preparations of the duodenum, ileum, and pelvic flexure from healthy horses concentration dependently [23,24]. Metoclopramide has proven to influence the intestinal motility of several animal species, including horses [25]. In the present study, the administration of metoclopramide did not show any significant effects on small intestinal motility (duodenum). This finding agreed with that observed following s/c administration of metoclopramide to Standardbred horses with the squamous gastric disease [1] and was supported by the fact that no evidence for the existence of 5-HT_4_ receptors in the equine jejunum is available [4,24]. By contrast, Okamura *et al*. [6] reported an increase in jejunal motility. Meanwhile, our data revealed a significant increase in cecal and colonic contractility 60 min following metoclopramide administration, which was also observed after 180 min. This finding agrees with that of a previous study, which showed the increased contractility of the pelvic flexure by a 5-HT_4_ agonist [23] and was supported by the successful role of metoclopramide in POI and colic countering [8]. By contrast, Okamura *et al*. [6] did not see any significant effect on the motility of the large intestine. This conflict may be due to variations in the density, distribution pattern, and expression of receptors in different parts of the equine GI tract [26]. Different dosages, administration frequencies, and routes of administration may also play an essential role in the literature variations [10]. Of interest, no behavioral abnormalities or other adverse reactions were observed in Arabian horses injected with metoclopramide. The previous studies, conversely, reported agitation, tremor, mild depression, and falling in some cases [3,6]. This discrepancy may be attributed to differences in metoclopramide dosage or horse breed.

## Conclusion

We concluded that although metoclopramide, as a prokinetic medication, only improves cecal and colonic contractions and not affecting the duodenum and that neostigmine improves duodenal, cecal, and colonic contractions, metoclopramide injection in healthy Arabian horses is safer and longer acting than neostigmine. Ultrasonography is a valuable non-invasive instrument for the quantification of intestinal contractility. To provide a more reliable conclusion, the complete pharmacokinetic profile of metoclopramide in equines warrants further studies with greater numbers of animals, including the evaluation of the effect of different dosages and routes of administration, besides its efficacy in clinical cases of GI motility disorders such as POI.

## Authors’ Contributions

NAB, MAA, and AAM conceived and designed the study. NAB, MAA, and AAM performed the study. NAB, MAA, and AAM analyzed the data. NAB and AAM wrote the paper. NAB, MAA, and AAM revised and approved the final manuscript. All authors read and approved the final manuscript.
